# Risk of Prediabetes and Diabetes in Oral Lichen Planus: A Case–Control Study according to Current Diagnostic Criteria

**DOI:** 10.3390/diagnostics13091586

**Published:** 2023-04-28

**Authors:** Lucía Rodríguez-Fonseca, Santiago Llorente-Pendás, María García-Pola

**Affiliations:** 1 Department of Surgery and Medical-Surgical Specialties, Faculty of Medicine and Sciences of the Health, Oviedo University, 33006 Oviedo, Spain; 2 Private Practice, 33004 Oviedo, Spain

**Keywords:** oral lichen planus, lichen planus, prediabetes, diabetes, oral antidiabetic, comorbidity

## Abstract

Objective: To estimate the prevalence of prediabetes and diabetes in patients with oral lichen planus (OLP). Methods: Prospective cohort, including consecutive patients diagnosed clinically and histologically with OLP from 2018 to 2022. Patients and controls were matched by age and gender. Fasting plasma glucose value collection from all patients. Multivariate regression analysis evaluated the relationship between prediabetes and diabetes variables according to current diagnostic criteria. Results: The sample comprised 275 patients (207 women; 75.3%), mean age 59.60 ± 12.18 years for both groups. Prediabetes was diagnosed according to the American Diabetes Association (ADA, 100–125 mg/dL), in 21.45% of OLP patients (59/275) and 14.55% (40/275) of control patients (*p* = 0.035). Patients with the atrophic-erosive form exhibited stronger association with taking oral antidiabetics (*p* = 0.011). Multivariate analysis showed that being over >60 years and having a cutaneous location was associated with ≥3 sites (OR 1.81 and OR 2.43). ADA prediabetes and oral antidiabetics drugs increased the probability of OLP (OR 1.60 (1.04–2.51), *p* = 0.03 and OR 2.20 (1.18–4.69), *p* = 0.017) after adjustment for sex and age. Conclusions: Because glycemia 100–125 mg/dL was associated with OLP, testing serum fasting plasma glucose seems reasonable in order to prevent development of diabetes and deal with possible complications until new studies are complete.

## 1. Introduction

Lichen planus (LP) is an inflammatory, mucocutaneous, chronic disease with periods of remission and exacerbation. LP affects the skin, nails, scalp, and mucous membranes, especially oral and genital [1]. Oral lichen planus (OLP) has a worldwide prevalence of 1.01%, is more common in women, and is increasingly common after the age of 40 [2].

Diagnosis is made by the presence of papular and reticular lesions with a more or less symmetrical location whether accompanied by any of the other clinical forms or not. It must be corroborated by microscopic study confirming the sub-basal inflammatory infiltrate, the liquefactive degeneration of the basal layer, and the absence of dysplasia [3]. OLP can present in association with other clinical forms, such as atrophic, erosive, or bullous forms. These non-keratotic forms appear more frequently with symptoms of burning or pain than keratotic forms such as papular, reticular or plaque-like [4,5].

Pathogenically, there is an immunological basis that is affected by various factors, to which oral commensal flora has recently been added [6]. In the oral inflammatory process, keratinocytes from OLP lesions produce proinflammatory cytokines. Additionally, in the pathogenic mechanism, the interaction of an antigen-presenting dendritic cell would initiate activation of cytotoxic CD8+ cells and CD4+ cells that differentiate into Th1 and Th17 cells. The complex protein cascade of cytokines, chemokines and adhesion molecules results in the promotion of apoptosis of keratinocytes and mucosal basement membrane destruction [7].

OLP is associated with viral infections and systemic comorbidities. In a general sense, Liu et al. highlighted the association with hepatitis C, diabetes mellitus (DM), thyroid diseases, dyslipidemias, cardiovascular diseases, and anxiety [8].

Diabetes is a heterogeneous group of diseases defined by chronically elevated blood glucose levels. The typical subclassification of diabetes includes type 1 diabetes (previously known as insulin-dependent, juvenile or childhood-onset diabetes), characterized by deficient insulin production in the body, and type 2 diabetes (non-insulin-dependent or adult-onset diabetes), resulting from the body’s ineffective use of insulin. Type 1 diabetes accounts for approximately 5–10% of all diabetes, whereas type 2 diabetes accounts for 90–95% [9]. The number of new cases of type 2 diabetes increases in the over 55s [10].

The pooled prevalence of DM amongst OLP patients has been indicated as 2.72%, with the hypothesis of being associated with autoimmune diseases such as DM type −1 [11]. A recent meta-analysis highlighted that the prevalence of diabetes in patients with OLP ranged from 1.62 for type 1 to 9.77 for non-specified diabetes [12]. That meta-analysis included a large number of varied study designs, including retrospective, prospective, cross-sectional, and longitudinal methodologies. Some studies collected data on diabetes through medical records, questionnaires, and interviews, while others had varying interpretations of diabetes based on the classic World Health Organization (WHO) criteria or the authors’ own criteria, among others.

Despite many studies having examined the prevalence of diabetes in OLP, only some reported statistically significant differences in glycemia levels compared to the normal population [13,14,15,16,17], while others found no differences [5,18,19,20,21,22,23]. Even fewer studies have indicated the link between OLP and prediabetes [24,25]. Therefore, our objective is to determine the prevalence and risk of prediabetes and diabetes in patients with OLP and control subjects according to the most current diagnostic criteria for prediabetes and diabetes based on serum parameters.

## 2. Materials and Methods

The present case–control study was designed according to the Strengthening Reporting of Observational Studies in Epidemiology (STROBE) guidelines (Supplementary Materials Table S1) [26]. The study was approved by the Ethics Committee of the Principality of Asturias as conforming to data protection regulation and the Declaration of Helsinki.

### 2.1. Patient Selection

The case group consisted of 275 OLP patients attending the Oral Medicine Unit at Oviedo University between January 2018 and November 2022. The sample size was calculated on the basis of a single proportion formula by the Epidat program with a 95% confidence interval (CI). Inclusion criteria for diagnosis of OLP were considered clinically and histologically. The clinical criteria included the presence of bilateral, mostly symmetrical lesions; the presence of a lace-like network of slightly raised gray-white lines in a reticular pattern; and by erosive, atrophic, bullous and plaque-type lesions [27]. Histopathological diagnosis by modified WHO criteria included: (1) well-defined band-like zones of cellular infiltration confined to the superficial part of the connective tissue; (2) “liquefactive degeneration” in the basal layer; and (3) the absence of epithelial dysplasia [3].

The protocol for clinical findings included: sex, age, daily tobacco and alcohol consumption, ex-smoker or ex-drinker, medical history, and prescription medication focusing on treatment of diabetes. The characteristics of OLP included a record of sites of involvement (cutaneous, genital, and scalp), predominant type of OLP (non-atrophic-erosive and atrophic-erosive), and number of sites (two sites or three or more sites).

The criteria for exclusion from the case group included: oral lichenoid lesion [28,29]; being under 18 years old; pregnant or breastfeeding women; and patients treated with topical or systemic corticosteroids, radiotherapy, or cancer chemotherapy.

The control group consisted of 275 consecutive patients who were examined in the same Oral Medicine Unit during the same period for other types of benign oral pathology-such as traumatic or benign tumors without any sign or history of OLP (n = 185) or for general oral health check-ups (n = 90). The controls were matched for age and sex to the patients with OLP.

Criteria for exclusion from the control group included: being under 18, pregnant or breastfeeding patients, and patients treated with radiotherapy or cancer chemotherapy.

### 2.2. Determination of Serum Parameters and Drugs

Glucose levels were examined in samples drawn before 10 a.m. after a 12 h fasting period. Serum parameters were collected after a first-day exploration of the mouth. The reference values for the parameters were based on: (1) the American Diabetes Association (ADA) prediabetes definition (100–125 mg/dL) [11]; (2) the WHO prediabetes definition (110–125 mg/dL) [12]; and (3) the WHO or ADA diabetes definition (≥126 mg/dL) [30,31].

Data was collected about drugs for treating diabetes, including insulin and oral antidiabetic drugs.

### 2.3. Statistical Analysis

**Figure 1:**
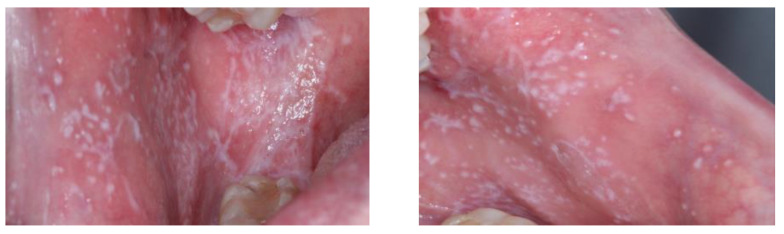
Clinical image of oral lichen planus of type non-atrophic-erosive located on both buccal mucosa.

**Figure 2:**
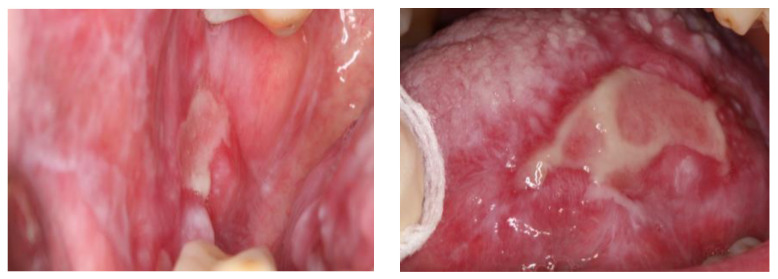
Clinical image of oral lichen planus of type atrophic-erosive located on buccal mucosa and lateral border of tongue.

Statistical analysis was done using R (R Development Core Team, version 4.1.3), and the information was recorded in an Excel file. Initially, we conducted a descriptive study of all of the variables. First, mean age was considered and subsequently split into two categories, selecting 60 years and under or over 60 years as break points. The predominant clinical form was classified as non-atrophic-erosive or atrophic-erosive (Figure 1 and Figure 2). The location of OLP was recorded as two locations or three or more locations. The ADA prediabetes, WHO prediabetes, WHO diabetes variables and medication were recorded as either present or absent. We added the variable type of treatment and recorded whether treatment was insulin-dependent or used antidiabetic drugs. Student’s *t*-test was used to compare the numerical variables between the two groups. The relationships between qualitative variables were assessed by Pearson’s chi-square test. The relationships were quantified by crude ratios and a multivariate logistic regression model produced using stepwise selection with variables whose *p* value was <0.20 in the univariate analysis and by age and sex. Results were considered statistically significant when *p* < 0.05.

## 3. Results

A total of 550 patients were analyzed (275 with OLP and 275 control patients). Both sample groups consisted of 207 women (75.27%) and 68 men (24.73%), and the average age was 59.60 ± 12.18 years (18–86). Table 1 shows the general characteristics of the case and control groups (Table 1).

Non-atrophic-erosive OLP was recorded in 98 patients (35.64%), and atrophic-erosive recorded in 177 (64.36%). Two intraoral sites were recorded in 139 patients (50.5%) and three or more sites were recorded in 136 (49.45%) patients. Forty-five patients (16.36%) exhibited extraoral manifestations.

The mean glucose level in the patients with OLP was 98.15 ± 21.32 and 95.45 ± 17.62 in the control group without statistically significant differences (*p* = 0.106). The prevalence of prediabetes according to ADA criteria was 21.45% (59/275) in the OLP group and 14.55% (40/275) in the control group [*p* = 0.035]. The prevalence of glycemic levels (≥126 mg/dL) was 8.73% (24/275) in the OLP group and 4.73% in the control group (13/275) [*p* = 0.061]. The prevalence of patients with treatment for diabetes was 11.64% (32/275) in the OLP group and 6.55% in the control group (18/275) [*p* = 0.038]. Therefore, despite receiving treatment, 15 (46.87%) of the 32 patients with diabetes still had a high glycemic level, as did 9 (50%) of the 18 subjects in the control group. A total of 9 (3.27%) patients with OLP and 4 (1.45%) from the control group did not know they were diabetics. The prevalence of patients with oral antidiabetic treatments was 10.18% (28/275) in the OLP group and 4.73% in the control group (13/275) [*p* = 0.015] (Table 1).

Table 2 shows the logistic regression analysis according to the clinical form (Table 2). The mean age of patients with atrophic-erosive forms of OLP was significantly higher (mean 60.97 yr; *p* = 0.014) than those with non-atrophic-erosive types (mean 57.15 yr), and in those over 60 years, the atrophic-erosive forms of OLP were more frequent (*p* = 0.006). There were no statistically significant differences in relation to clinical form or sex (*p* = 0.946) or location in genitals (*p* = 0.396), skin (*p* = 0.891) or scalp (*p* = 0.694).

There were statistically significant differences in relation to clinical form and oral antidiabetic treatment (*p* = 0.005), which was also higher in atrophic-erosive forms of OLP (26; 92.9%) than in non-atrophic-erosive types (2; 7.1%).

The logistic regression confirmed the statistically significant association of age >60 years (*p* = 0.007) and oral antidiabetic treatments (*p* = 0.021) with the atrophic-erosive clinical form. In patients who were over 60, there was also a statistically significant difference in terms of sites (*p* = 0.036), with three or more sites of OLP (74; 56.1%) being more common than two (58; 43.9%).

The logistic regression confirmed the statistically significant association of WHO prediabetes (*p* = 0.015) with two sites (Table 3). Nevertheless, the multivariate study determined that there was a higher probability of ≥3 sites being affected at >60 years (OR 1.81; *p* = 0.018) and in patients with cutaneous involvement (OR 2.43; *p* = 0.036).

The results of the logistic regression analyses with regard to glycemia, prediabetes and diabetes are shown in Table 4 (Table 4). In the univariate study, patients with ADA prediabetes and those taking oral antidiabetics were more likely to be suffering from OLP, and in the multivariate analysis adjusted for sex and age (≤60), this was corroborated (OR_ADAprediabetes_ = 1.61 (1.04–2.51) and OR_oral antidiabetic_ = 2.29 (1.18–4.68)).

## 4. Discussion

The present study examined a sample of people with OLP whose fasting plasma glucose values and presence of DM were analyzed and compared to a control group. The initial interpretation of our results indicated that prediabetes with glycemia from 100 mg/dL to 125 mg/dL and intake of oral antidiabetics were associated with OLP, which was confirmed when adjusted for sex and age.

There were no statistically significant differences in mean glycemic levels between OLP cases (98.15 mg/dL) and the control group (95.45 mg/dL) as previously reported by Lopez et al. [32]. Although those authors recorded higher levels in the control group (n = 200; 102.5 ± 76.8) than in OLP patients (n = 200; 101.2 ± 22), they found no statistically significant differences [32].

Various case–control studies have not found an association between OLP and DM, but they all failed to note the diagnostic criteria for diabetes [5,18,19,20,21,22,23]. Other studies have reported statistically significant differences, with DM being more prevalent in OLP patients than in control groups according to medical records [13,14], or electronic medical records [15].

Previous studies have referred to a diagnosis of DM for blood glucose values ≥100 mg/dL, that is, prediabetes and diabetes [24,25]. In our results, the proportion of patients with values greater than ≥100 mg/dL (30.18%) is similar to the figures those studies reported for OLP groups of 30.11% [24] and 35% [25] with samples of 177 and 40 patients, respectively.

The prevalence of blood glucose levels > 125 mg/dL in previous studies with 40 OLP patients has been reported as 28% [16], whereas in our study, 8.73% of the OLP patients had levels ≥ 126 mg/dL. More recently, Chalkoo considered DM when fasting blood glucose >120 mg/dL, reporting a prevalence of DM of 35% in 20 OLP patients, with statistically significant differences compared to the control group [17].

Chung et al. [33], studied treatments for DM using questionnaires and found statistically significantly higher levels of treatment for DM in 32 patients with OLP compared to the control group. From a sample of 156 OLP and 156 controls, Dave et al. [14]. reported statistically significant differences in treatment with biguanides and insulin but not with sulfanyl ureas. In their multivariate model, only DM appeared as a risk factor (OR, 2.8), not treatments for DM. We confirmed that once adjusted for sex and age, the association between oral antidiabetics and OLP was twice as strong as in the control group.

A relationship between the reticular clinical form and treatment of DM in patients with OLP has been described [33], as has a relationship between atrophic-erosive lesions and DM treatment [34]. Our findings confirmed an association between the atrophic-erosive clinical form and oral antidiabetics, and age > 60 years. Although other authors have not found these relationships between the clinical form and DM, Zhao et al. observed that after treatment of erosive OLP with hyperglycaemia, glycemic levels fell [35].

Logistic regression showed that none of the parameters of the diabetes variables were associated with the number of sites of OLP. Instead, being over 60 and having skin involvement were associated with greater increases in the risk of presenting OLP (OR, 1.81, and OR, 2.43, respectively)–risks that we have not seen reported in the literature.

It is interesting to note from our results that between 1.45% (control group) and 3.27% (OLP patients) of the subjects were unaware of having DM. Between 5–10% of patients per year with impaired fasting glucose or impaired glucose tolerance can develop type 2 diabetes, hence the recommendation to screen for diabetes in people who are 35 and over and to test for it annually. The term prediabetes is used to label an elevated glucose level that does not meet the criteria for diabetes, and the ADA recommends identification of individuals with prediabetes, providing an opportunity for intervention [11]. Despite the fact that the progression of OLP does not influence average glucose levels, OLP is a chronic disease, and patients may develop prediabetes or progress from prediabetes to diabetes. Our findings indicate the need to establish preventive measures such as lifestyle modification in patients with OLP to avoid progressing to diabetes. This need is even greater if corticosteroids are used as the first choice of treatment for OLP [36], as they are absorbed following topical application [37], and their adverse effects—hyperglycemia and diabetes—are greater where there is comorbidity [38].

The possible link between diabetes and OLP comes from the presence of common histocompatibility antigens (HLA), especially HLA28, in both diseases [39]. More recently, higher levels of serum interleukins 8 have been observed in OLP and diabetes independently and where they are associated with each other, which would support the relationship between them [40]. Furthermore, the importance of the topic is reinforced from the bidirectional point of view of oral cancer, assuming the association of OLP and oral cancer in patients with diabetes, and oral cancer in patients with OLP [11].

The present study has some limitations. This was a single-center study with a cross-sectional design, so it does not allow causality or pathogenic association to be inferred from our results. Another limitation to consider is that the study did not include variables that could be related to lifestyle or other metabolic diseases associated with DM; however, it does provide interesting future lines for research to confirm the relationship between prediabetes and OLP.

## 5. Conclusions

In conclusion, we found significantly higher frequencies of glycemia in the order of 100–125 mg/dL in OLP patients. It therefore seems reasonable to test serum fasting plasma glucose values in order to prevent the development of diabetes as well as deal with its possible complications. Our results should be corroborated by new studies to avoid complications of prediabetes in OLP patients.

## Figures and Tables

**Table 1:** Characteristics of patients from control group and oral lichen planus group (OLP). Numbers in parentheses represent percentages. ^1^: In addition to oral location. ADA (American Diabetes Association) (100–125 mg/dL). WHO prediabetes: World Health Organization (110–125 mg/dL). *: Statistically significant.

Variable		OLP	Control	*p* Value
Sex	Female	207 (75.27)	207 (75.27)	1
	Male	68 (24.73)	68 (24.73)	
Age	Mean (SD)	59.61 (12.19)	59.61 (12.19)	1
	≤60	143 (52.0)	143 (52.0)	1
	>60	132 (48.0)	132 (48.0)	
Tobacco user	No	203 (73.82)	193 (70.18)	0.09
	Yes	28 (10.18)	45 (16.36)	
	Ex-smoker	44 (16.0)	37 (13.45)	
Alcohol drinker	No	221 (80.36)	223 (81.09)	0.956
	Yes	52 (18.91)	52 (18.91)	
	Ex-drinker	2 (0.73)		
Clinical-form OLP				
	Non-atrophic-erosive	98 (35.64)		
	atrophic-erosive	177 (64.36)		
Oral location	2	246 (89.45)		
	≥3	29 (10.55)		
Cutaneous ^1^	No	246 (89.45)		
	Yes	29 (10.55)		
Genital ^1^	No	253 (92.0)		
	Yes	22 (8.0)		
Scalp ^1^	No	268 (97.45)		
	Yes	7 (2.55)		
Glycemia	(mean, mg/dL (SD)	95.45 ± 17.62	98.15 ± 21.32	0.106
ADA prediabetes	No	235 (85.45)	216 (78.55)	0.035 *
	Yes	40 (14.55)	59 (21.45)	
WHO prediabetes	No	253 (92.0)	259 (94.18)	0.313
	Yes	22 (8.0)	16 (5.82)	
Diabetes (≥126 mg/dL)	No	262 (95.27)	251 (91.27)	0.061
	Yes	13 (4.73)	24 (8.73)	
Treatment of diabetes	No	257 (93.45)	243 (88.36)	0.038 *
	Yes	18 (6.55)	32 (11.64)	
Type of treatment	Insulin	5 (1.82)	4 (1.45)	0.015 *
	Oral antidiabetic	13 (4.73)	28 (10.18)	

**Table 2:** Statistically significant associations (*p <* 0.05 *) according to the logistic regression by clinical form. OR: Odd ratio. CI: confidence interval. Percentages in parentheses. ADA prediabetes: American Diabetes Association (100–125 mg/dL). WHO prediabetes: World Health Organization (110–125 mg/dL). ^1^: In addition to oral location. SD: standard deviation.

Variable	Non-Atrophic-Erosive	Atrophic-Erosive	OR Univariate (CI, *p* Value)	OR Multivariate (CI, *p* Value)
Sex				
Female	74 (35.7)	133 (64.3)		
Male	24 (35.3)	44 (64.7)	0.98 (0.55–1.73, *p* = 0.946)	
Mean age (SD)	57.15 (11.68)	60.97 (12.39)	0.97 (0.95–0.99, *p* = 0.014) *	-
Age ≤ 60 years				
≤60 years	62 (43.4)	81 (56.6)		
>60 years	36 (27.3)	96 (72.7)	0.49 (0.29–0.81, *p* = 0.006) *	0.54 (0.32–0.91, *p* = 0.021) *
Genitals ^1^				
No	92 (36.4)	161 (63.6)		
Yes	6 (27.3)	16 (72,7)	0.66 (0.23–1.66, *p* = 0.396)	-
Cutaneous ^1^				
No	88 (35.8)	158 (64.2)		
Yes	10 (34.5)	19 (65.5)	0.94 (0.41–2.08, *p* = 0.891)	-
Scalp ^1^				
No	96 (35.8)	172 (64.2)		
Yes	2 (28.6)	5 (71.4)	0.72 (0.10–3,39, *p* = 0.694)	-
Glycemia (mean)	95.2 (19.9)	99.8 (22.0)	0.99 (0.97–1.00, *p* = 0.097)	1.02 (1.00–1.05, *p*= 0.053)
ADA prediabetes				
No	81 (37.5)	135 (62.5)		
Yes	17 (28.8)	42 (71.2)	0.67 (0.35–1.24, *p* = 0.219)	
WHO prediabetes				
No	93 (35.9)	166 (64.1)		
Yes	5 (31.2)	11 (68.8)	0.81 (0.25–2.30, *p* = 0.706)	-
Diabetes (≥126 mg/dL)				
No	91 (36.3)	160 (63.7)		
Yes	7 (29.2)	17 (70.8)	0.72 (0.27–1.75, *p* = 0.490)	2.51 (0.76–8.64, *p* = 0.131)
Type of treatment No Oral antidiabetics Insulin	95 (39.1) 2 (7.1) 1 (25.0)	148 (60.9) 26 (92.9) 3 (75.0)	0.12 (0.02–0.42, *p* = 0.004) * 0.52 (0.03–4.12, *p* = 0.573)	0.17 (0.03–0.62, *p* = 0.011) * 0.64 (0.03–5.44, *p* = 0.704)

**Table 3:** Statistically significant associations (*p <* 0.05 *) according to the logistic regression by number of locations. OR: Odd ratio. CI: confidence interval. Percentages in parentheses. ADA: American Diabetes Association criteria. WHO: World Health Organization. ^1^: In addition to oral location. SD: standard deviation.

Variable	2 Sites	≥3 Sites	OR Univariate (CI, *p* Value)	OR Multivariate (CI, *p* Value)
Sex				-
Female	102 (49.3)	105 (50.7)		
Male	37 (54.4)	31 (45.6)	0.81 (0.47–1.41, *p* = 0.463)	
Mean age (SD)	58.5 (12.68)	60.8 (12.0)	1.02 (1.00–1.04, *p* = 0.121)	-
Age ≤ 60 years				
≤60 years	81 (56.6)	62 (43.4)		
>60 years	58 (43.9)	74 (56.1)	1.67 (1.04–2.69, *p* = 0.036) *	1.81 (1.11–2.97, *p* = 0.018) *
Genitals ^1^				
No	129 (51.0)	124 (49.0)		
Yes	10 (45.5)	12 (54.5)	1.25 (0.52–3.06, *p* = 0.619)	-
Cutaneous ^1^				
No	129 (52.4)	117 (47.6)		
Yes	10 (34.5)	19 (65.5)	2.09 (0.95–4.86, *p* = 0.072)	2.43 (1.04–5.78, *p* = 0.036) *
Scalp ^1^				
No	136 (50.7)	132 (64.2)		
Yes	3 (42.9)	4 (57.1)	1.37 (0.30–7.08, *p* = 0.681)	-
Glycemia (mean)	97.4 (22.2)	99.8 (20.4)	1.00 (0.99–1.02, *p* = 0.543)	-
ADA prediabetes				
No	112 (51.9)	104 (48.1)		
Yes	27 (45.8)	32 (54.2)	1.28 (0.72–2.29, *p* = 0.408)	-
WHO prediabetes				
No	126 (48.6)	133 (51.4)		
Yes	13 (81.2)	3 (18.8)	0.22 (0.05–0.70, *p* = 0.020) *	0.20 (0.04–0.65, *p* = 0.015) *
Diabetes (≥126 mg/dL)				
No	126 (50.2)	125 (49.8)		
Yes	13 (54.2)	11 (45.8)	0.85 (0.36–1.98, *p* = 0.711)	-
Type of treatment No Oral antidiabetics Insulin	124 (51.0) 12 (42.9) 3 (75.0)	119(49.0) 16 (57.19) 1 (25.0)	1.41 (0.64–3.17, *p* = 0.392) 0.35 (0.02–2.76, *p* = 0.363)	

**Table 4:** Association between oral lichen planus and glycemic level adjusted for age (≤60) and sex. Statistically significant associations (*p <* 0.05 *). OR: Odd ratio. CI: confidence interval. ADA: American Diabetes Association. WHO: World Health Organization.

Variable		OLP	Control	OR Univariate	OR Multivariate
ADA prediabetes (100–125 mg/dL)	No Yes	216 (47.9) 59 (59.6)	235 (52.1) 40 (40.4)	1.60 (1.03–2.51) *p* = 0.036 *	1.61 (1.04–2.51) *p* = 0.036 *
WHO prediabetes (110–125 mg/dL)	No Yes	259 (50.6) 16 (42.1)	253 (49.4) 22 (57.9)	0.71 (0.36–1.38) *p* = 0.315	0.71 (0.36–1.38) *p* = 0.315
Diabetes (≥126 mg/dL)	No Yes	251 (48.9) 24 (64.9)	262 (51.1) 13 (35.1)	1.93 (0.97–3.98) *p* = 0.065	1.96 (0.98–4.08) *p* = 0.062
Oral antidiabetics	No Yes	247 (48.5) 28 (68.3)	262 (51.1) 13 (31.7)	2.28 (1.18–4.65) *p* = 0.017 *	2.29 (1.18–4.68) *p* = 0.017 *

## Data Availability

The data presented in this study are available on request from the corresponding author.

## Supplementary Materials

The following supporting information can be downloaded at: https://www.mdpi.com/article/10.3390/diagnostics13091586/s1, Table S1: STROBE Statement—Checklist of items that should be included in reports of case-control studies.

## References

[1] Le Cleach L., Chosidow O. (2012). Lichen planus. N. Engl. J. Med..

[2] González-Moles M.Á., Warnakulasuriya S., González-Ruiz I., González-Ruiz L., Ayén Á., Lenouvel D., Ruiz-Ávila I., Ramos-García P. (2021). Worldwide prevalence of oral lichen planus: A systematic review and meta-analysis. Oral Dis..

[3] van der Meij E.H., van der Waal I. (2003). Lack of clinicopathologic correlation in the diagnosis of oral lichen planus based on the presently available diagnostic criteria and suggestions for modifications. J. Oral Pathol. Med..

[4] Carbone M., Arduino P.G., Carrozzo M., Gandolfo S., Argiolas M.R., Bertolusso G., Conrotto D., Pentenero M., Broccoletti R. (2009). Course of oral lichen planus: A retrospective study of 808 northern Italian patients. Oral Dis..

[5] Adamo D., Calabria E., Coppola N., Lo Muzio L., Giuliani M., Bizzoca M.E., Azzi L., Croveri F., Colella G., Boschetti C.E. (2021). SIPMO (Italian Society of Oral Pathology, Medicine). Psychological profile and unexpected pain in oral lichen planus: A case-control multicenter SIPMO study. Oral Dis..

[6] De Angelis L.M., Cirillo N., McCullough M.J. (2019). The immunopathogenesis of oral lichen planus-Is there a role for mucosal associated invariant T cells?. J. Oral Pathol. Med..

[7] El-Howati A., Thornhill M.H., Colley H.E., Murdoch C. (2022). Immune mechanisms in oral lichen planus. Oral Dis..

[8] Liu W., Deng Y., Shi H., Shen X. (2022). Clinical investigation on oral lichen planus and associated comorbidities needs a holistic concept. Oral Dis..

[9] American Diabetes Association Professional Practice Committee (2022). Classification and Diagnosis of Diabetes: Standards of Medical Care in Diabetes. Diabetes Care.

[10] Ahmad E., Lim S., Lamptey R., Webb D.R., Davies M.J. (2022). Type 2 diabetes. Lancet.

[11] Ramos-Garcia P., Roca-Rodriguez M.D.M., Aguilar-Diosdado M., Gonzalez-Moles M.A. (2021). Diabetes mellitus and oral cancer/oral potentially malignant disorders: A systematic review and meta-analysis. Oral Dis..

[12] De Porras-Carrique T., Ramos-García P., Aguilar-Diosdado M., Warnakulasuriya S., González-Moles M.Á. (2022). Autoimmune disorders in oral lichen planus: A systematic review and meta-analysis. Oral Dis..

[13] Canjuga I., Mravak-Stipetić M., Lončar B., Kern J. (2010). The Prevalence of Systemic Diseases and Medications in Patients with Oral Lichen Planus. Acta Stomatol. Croat..

[14] Dave A., Shariff J., Philipone E. (2021). Association between oral lichen planus and systemic conditions and medications: Case-control study. Oral Dis..

[15] Arduino P.G., Karimi D., Tirone F., Sciannameo V., Ricceri F., Cabras M., Gambino A., Conrotto D., Salzano S., Carbone M. (2017). Evidence of earlier thyroid dysfunction in newly diagnosed oral lichen planus patients: A hint for endocrinologists. Endocr. Connect..

[16] Lundström I.M. (1983). Incidence of diabetes mellitus in patients with oral lichen planus. Int. J. Oral Maxillofac. Surg..

[17] Chalkoo A.H. (2010). Oral Lichen Planus: Relation with Transaminase Levels and Diabetes. Oral Lichen Planus: Relation with Transaminase Levels and Diabetes. J. Indian Acad. Oral Med. Radiol..

[18] Garg V.K., Karki B.M., Agrawal S., Agarwalla A., Gupta R. (2002). A study from Nepal showing no correlation between lichen planus and hepatitis B and C viruses. J. Dermatol..

[19] Gerayli S., Meshkat Z., Pasdar A., Mosannen Mozafari P., Banihashemi E., Khajavi M.A., Rasekhi J. (2015). The association between oral lichen planus and hepatitis C virus infection; a report from northeast of Iran. Jundishapur. J. Microbiol..

[20] Kats L., Goldman Y., Kahn A., Goldman V., Gorsky M. (2019). Oral lichen planus and thyroid gland diseases: Possible associations. BMC Oral Health.

[21] Nagao Y., Sata M. (2012). A retrospective case-control study of hepatitis C virus infection and oral lichen planus in Japan: Association study with mutations in the core and NS5A region of hepatitis C virus. BMC Gastroenterol..

[22] Xue J.L., Fan M.W., Wang S.Z., Chen X.M., Li Y., Wang L. (2005). A clinical study of 674 patients with oral lichen planus in China. J. Oral Pathol. Med..

[23] Zhou T., Li D., Chen Q., Hua H., Li C. (2018). Correlation Between Oral Lichen Planus and Thyroid Disease in China: A Case-Control Study. Front. Endocrinol..

[24] Hornstein O.P., Stühler C., Schirner E., Simon M. (1984). Lichen ruber and diabetes mellitus—Pathogenetic relations?. Hautarzt.

[25] Ali A.A., Suresh C.S. (2007). Oral lichen planus in relation to transaminase levels and hepatitis C virus. J. Oral Pathol. Med..

[26] Vandenbroucke J.P., von Elm E., Altman D.G., Gøtzsche P.C., Mulrow C.D., Pocock S.J., Poole C., Schlesselman J.J., Egger M. (2014). Strengthening the Reporting of Observational Studies in Epidemiology (STROBE): Explanation and elaboration. Int. J. Surg..

[27] Kramer I.R., Lucas R.B., Pindborg J.J., Sobin L.H. (1978). Definition of leukoplakia and related lesions: An aid to studies on oral precancer. Oral Surg. Oral Med. Oral Pathol..

[28] Aguirre-Urizar J.M., Alberdi-Navarro J., Lafuente-Ibáñez de Mendoza I., Marichalar-Mendia X., Martínez-Revilla B., Parra-Pérez C., De Juan-Galindez A., Echebarria-Goicouria M.Á. (2020). Clinicopathological and prognostic characterization of oral lichenoid disease and its main subtypes: A series of 384 cases. Med. Oral Patol. Oral Cir. Bucal..

[29] van der Waal I. (2009). Oral lichen planus and oral lichenoid lesions; a critical appraisal with emphasis on the diagnostic aspects. Med. Oral Patol. Oral Cir. Bucal..

[30] (2018). American Diabetes Association. Standards of Medical Care in Diabetes-2018 Abridged for Primary Care Providers. Clin. Diabetes.

[31] World Health Organization (2016). Global Report on Diabetes.

[32] López-Jornet P., Camacho-Alonso F., Rodríguez-Martínes M.A. (2012). Alterations in serum lipid profile patterns in oral lichen planus: A cross-sectional study. Am. J. Clin. Dermatol..

[33] Chung C.H., Yang Y.H., Chang T.T., Shieh D.B., Liu S.Y., Shieh T.Y. (2004). Relationship of oral lichen planus to hepatitis C virus in southern Taiwan. Kaohsiung J. Med. Sci..

[34] Bagan J.V., Donat J.S., Penarrocha M., Milian M.A., Sanchis J.M. (1993). Oral lichen planus and diabetes mellitus. A clinico-pathological study. Bull. Group Int. Rec. Sci. Stomatol. Odontol..

[35] Zhao W., Yang Y., Wu F., Zhou H. (2019). The reciprocal association between diabetes mellitus and erosive oral lichen planus. Oral Dis..

[36] Thongprasom K. (2018). Oral lichen planus: Challenge and management. Oral Dis..

[37] Varoni E.M., Molteni A., Sardella A., Carrassi A., Di Candia D., Gigli F., Lodi F., Lodi G. (2012). Pharmacokinetics study about topical clobetasol on oral mucosa. J. Oral Pathol. Med..

[38] Ceccarelli E., Mattaliano C., Brazzi A., Marinetti A.C., Nigi L., Chirico C., Corallo C., Fioravanti A., Giordano N. (2018). Hyperglycemia and diabetes induced by glucocorticoids in nondiabetic and diabetic patients: Revision of literature and personal considerations. Curr. Pharm. Biotechnol..

[39] Halevy S., Zamir R., Gazit E., Feuerman E.J. (1979). HLA system in relation to carbohydrate metabolism in lichen planus. Br. J. Dermatol..

[40] Tavangar A., Khozeimeh F., Ghoreishian F., Boroujeni M.A. (2016). Serum level of Interleukin-8 in subjects with diabetes, diabetes plus oral lichen planus, and oral lichen planus: A biochemical study. Dent. Res. J..

